# Supplementary figures and images for: Characterization of CRISPR/Cas9 RANKL knockout mesenchymal stem cell clones based on single-cell printing technology and Emulsion Coupling assay as a low-cellularity workflow for single-cell cloning
Source: PLoS One. 2021 Mar 4;16(3):e0238330. doi: 10.1371/journal.pone.0238330 (PMC7932140; doi:10.1371/journal.pone.0238330)

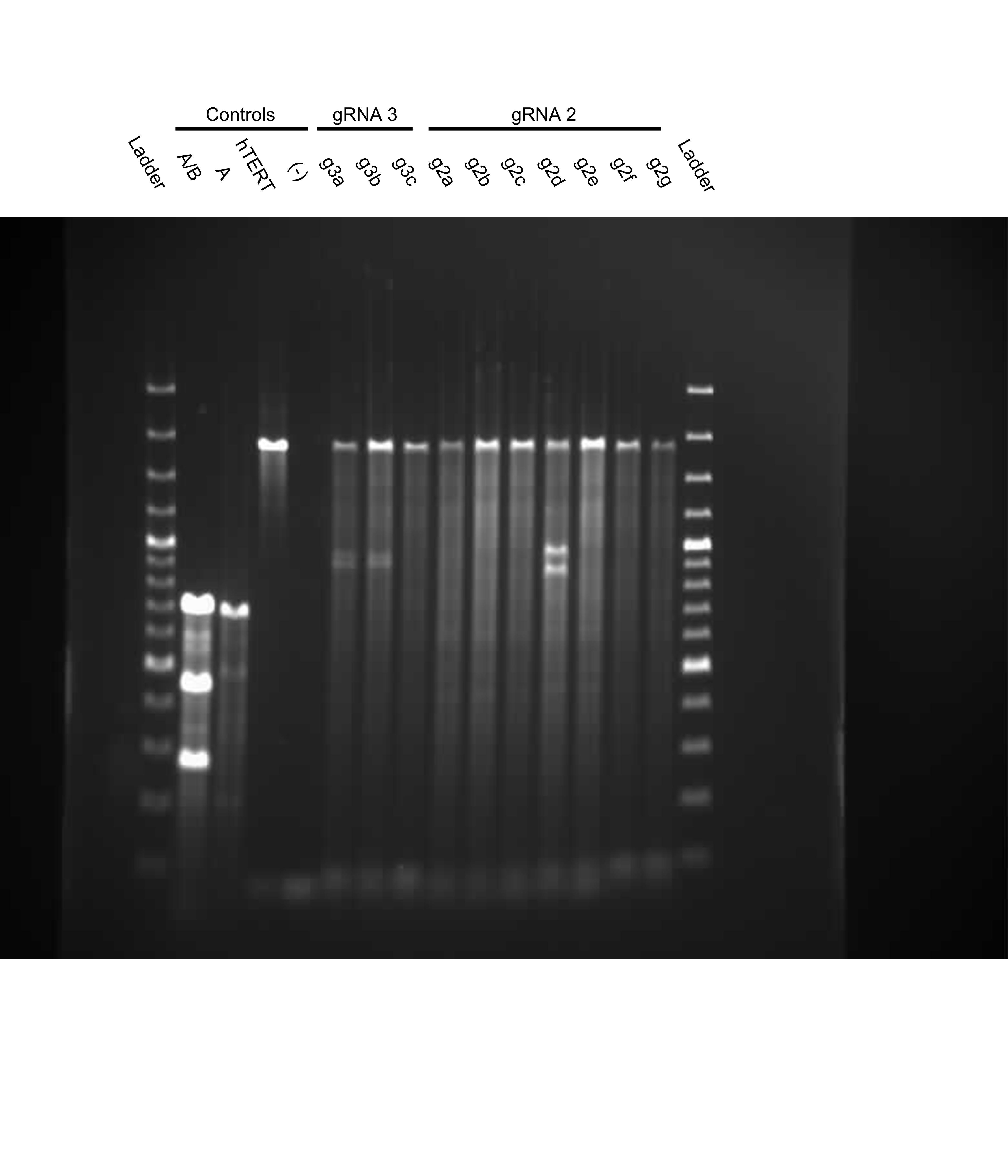

Supplement: S1 Raw image — (TIF) [file pone.0238330.s001.tif]

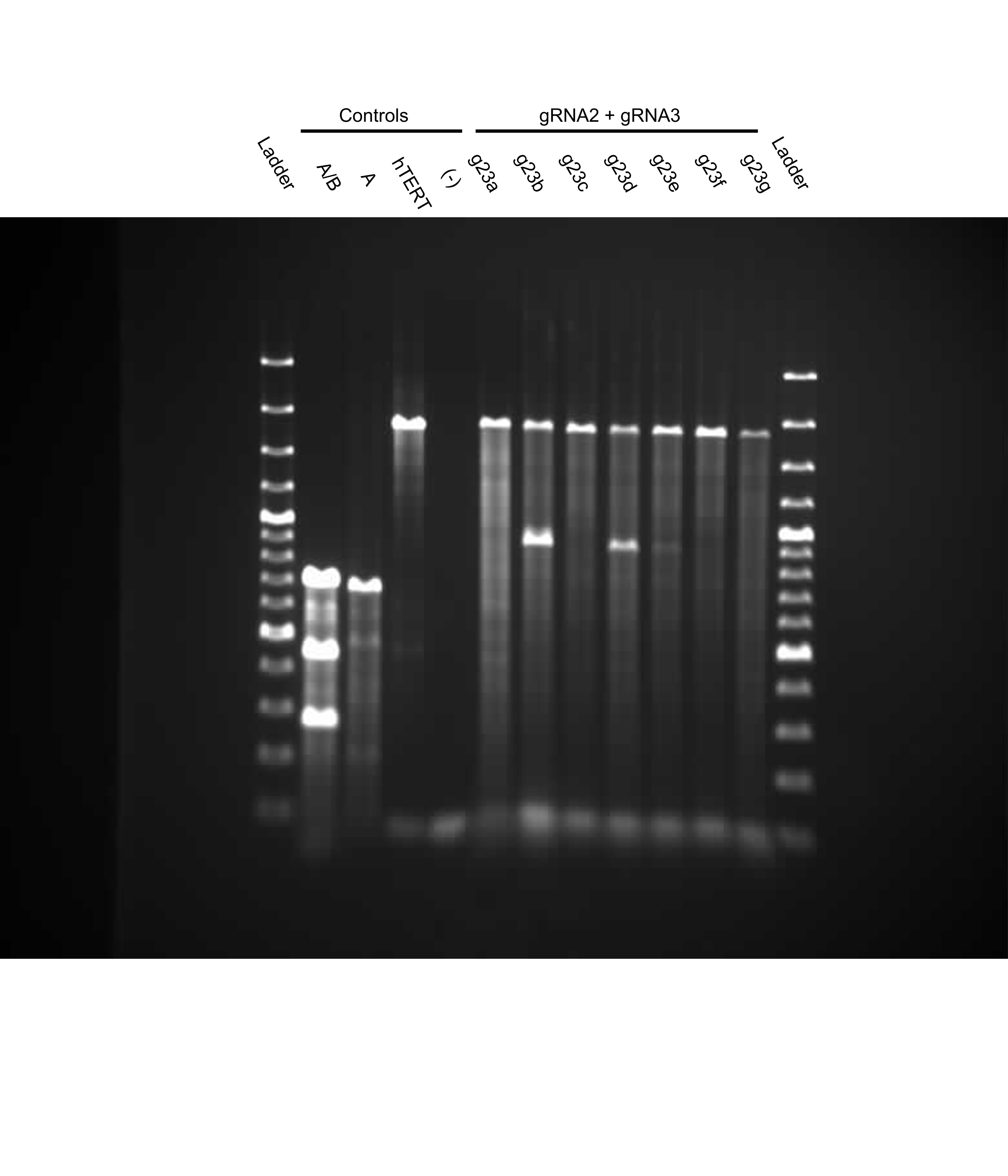

Supplement: S2 Raw image — (TIF) [file pone.0238330.s002.tif]
